# Intramedullary spinal cord abscess with brain abscess due to subacute infective endocarditis

**DOI:** 10.1186/s12883-023-03050-8

**Published:** 2023-01-16

**Authors:** Weigang Luo, Yuanyuan Yin, Wanhu Liu, Huiling Ren

**Affiliations:** Department of Neurology, The Third Hospital of Hebei Medical University, Shijiazhuang, China

**Keywords:** Intramedullary spinal cord abscess, Brain abscess, *Streptococcus anginosus*, Infective endocarditis, Case report

## Abstract

**Background:**

Intramedullary spinal cord abscesses (ISCA) are rare, even more so in association with brain abscesses. Infective endocarditis is an uncommon cause of ISCA. In this case study, we report a patient with intramedullary abscesses and multiple brain abscesses due to subacute infective endocarditis.

**Case presentation:**

A 54-year-old man presented with a 7-day history of head and neck pain and numbness in both lower limbs. Intramedullary abscess combined with multiple brain abscesses was diagnosed based on blood culture, head and spinal magnetic resonance imaging (MRI), contrast-enhanced MRI, and magnetic resonance spectroscopy. Echocardiography revealed vegetations on the mitral valve and severe mitral regurgitation, which the authors believe was caused by subacute infective endocarditis. With ceftriaxone combined with linezolid anti-infective therapy, the patient's symptoms and imaging was improved during follow-up.

**Conclusions:**

This case hopes to raise the vigilance of clinicians for ISCA. When considering a patient with an ISCA, it is necessary to complete blood culture, MRI of the brain and spinal cord, and echocardiography to further identify whether the patient also has a brain abscess and whether the cause is infective endocarditis.

## Introduction

Intramedullary spinal cord abscess (ISCA) is a rare infection of the central nervous system, and it is even rarer in patients with brain abscesses. Normal spinal cord tissue is remarkably resistant to infection, and ISCA commonly occurs when patients have one of the following four specific categories of the underlying disease: bacterial and fungal infection, penetrating trauma to the spinal cord, congenital dural sinuses or chronic tuberculosis [1]. Among them, the infection focus was infective endocarditis, accounting for 5% [2]. ISCA is characterized by progressive back pain and neurological deficits, but clinical manifestations can also progress insidiously [3]. Spinal cord intramedullary abscesses have rapidly progressive symptoms, high mortality,and poor prognosis [4]. Therefore, early diagnosis and timely treatment are crucial for preventing further neurological decline and reducing mortality. To improve clinicians' awareness and vigilance of intramedullary abscesses, we report here a case of an ISCA with multiple brain abscesses due to subacute infective endocarditis.

## Case report

A 54-year-old man presented with a 7-day history of head and neck pain and numbness in both lower limbs for 7 days. In the 10 days before admission, the patient presented with blurred vision, occasional blindness in both eyes and mild neck pain. Three days after the onset of symptoms, the patient developed headache, progressive neck pain, numbness in both lower limbs, and numbness in the tips of both fingers. There is no limb weakness, no fever and night sweats, no bladder and bowel dysfunction. The patient denied any recent weight loss, furuncles, coughs, odontogenic lesions, and presented to a local hospital. Magnetic resonance imaging (MRI) showed high signal intensity on T2WI of C2-C7 segments and mixed signals behind the right intracranial ventricle. For further diagnosis and treatment, he was admitted to our hospital.

**Fig. 1:**
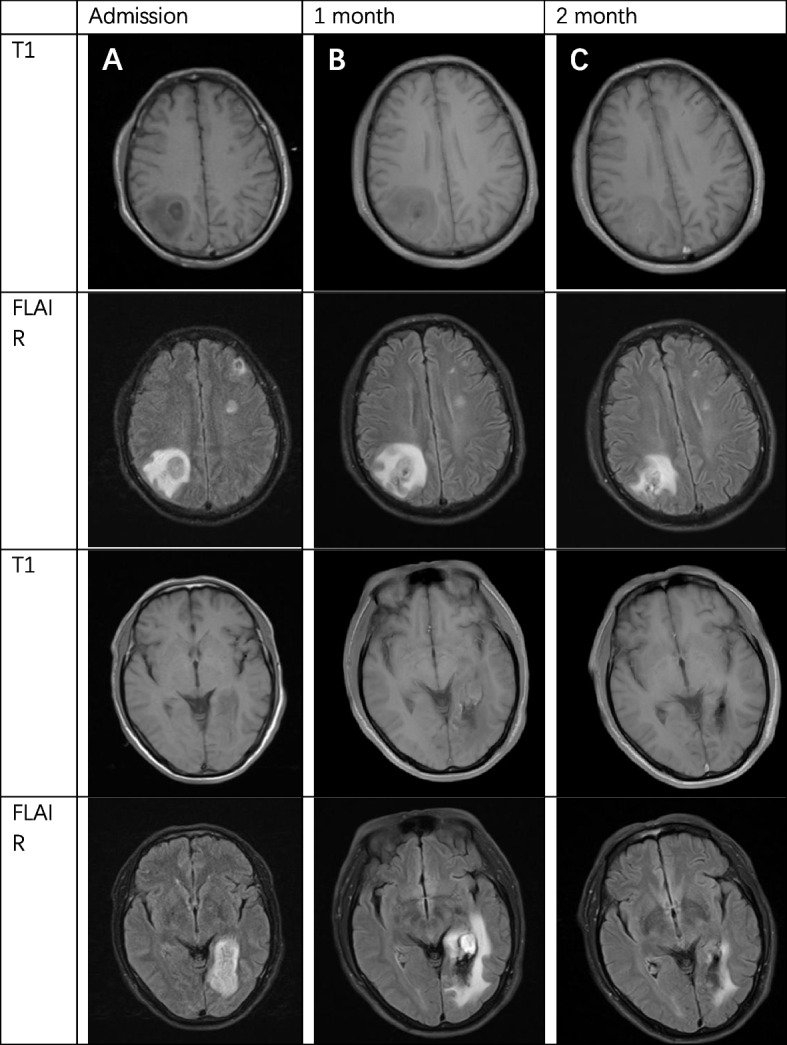
MRI of brain abscess changes during treatment. **A-C** (from onset to 2 months) showed progressive reduction of cerebral lesions with prolonged treatment

**Fig. 2:**
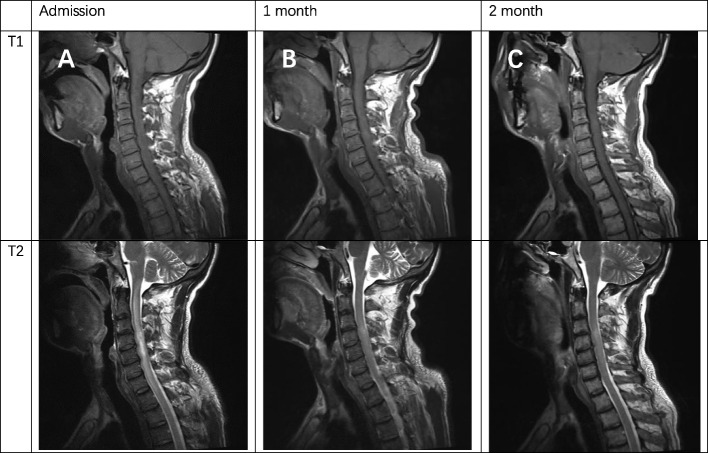
MRI of spinal cord abscess changes during treatment. **A-C** (from onset to 2 months) showed progressive reduction of cerebral lesions with prolonged treatment

**Fig. 3:**
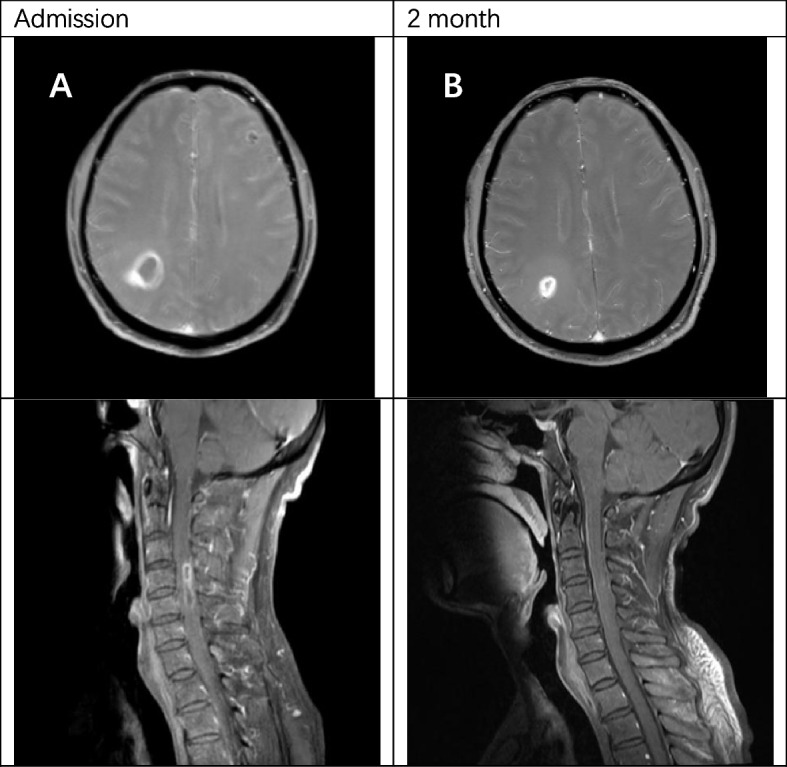
Comparison of brain and spinal cord enhancement images before and after treatment. **A** The post-T1 contrast images of the brain and spinal cord at admission showed continuous enhancement of the peripheral ring. **B** The ring enhancement of the brain and spinal cord lesions became smaller after 2 months of treatment

**Fig. 4:**
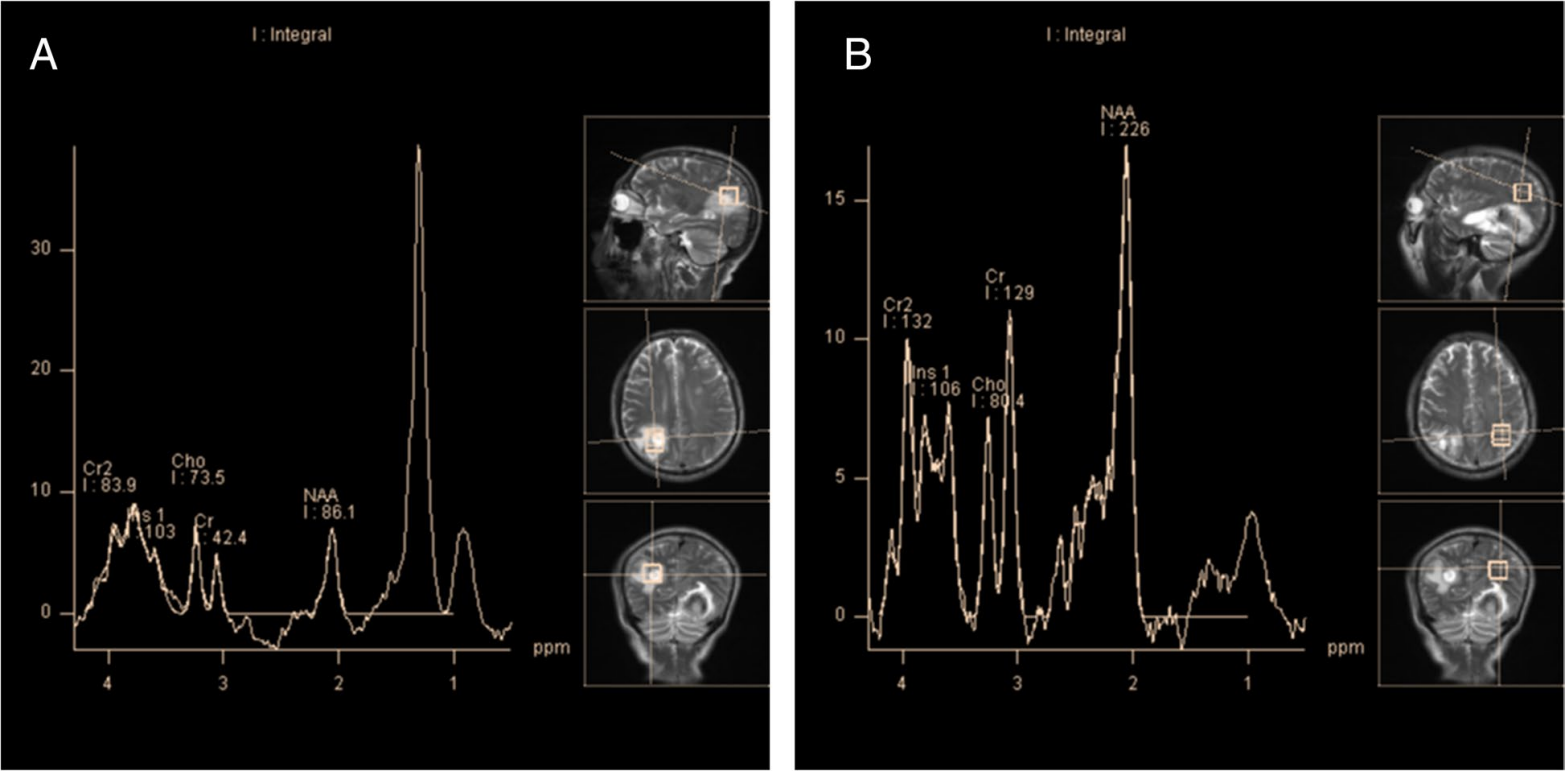
Magnetic resonance spectroscopy of bilateral parietal lobes. **A** Magnetic resonance spectroscopy of the parietal lobe at the lesion side. **B** Magnetic resonance spectroscopy of contralateral parietal lobe

**Fig. 5:**
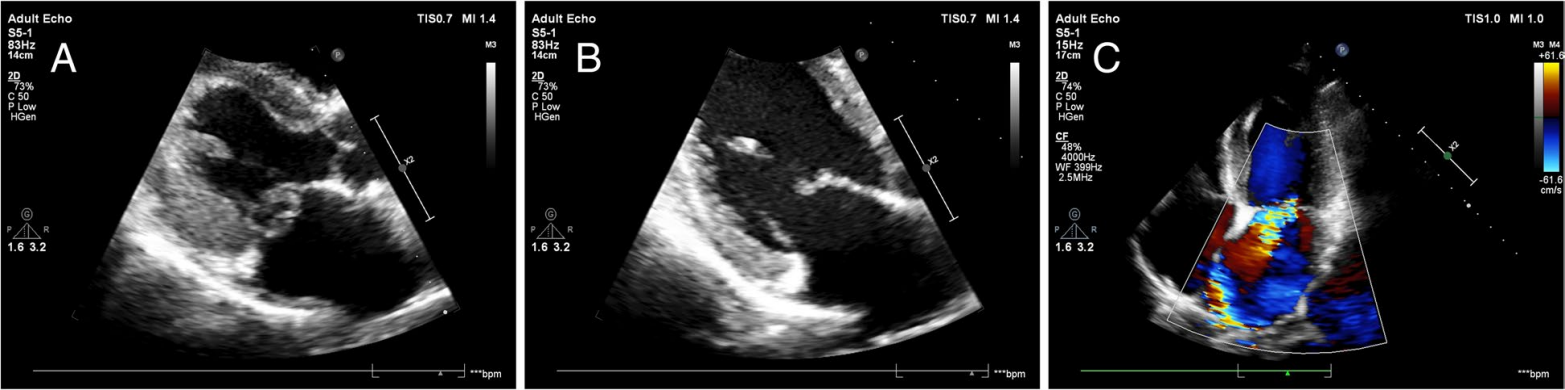
Echocardiogram. **A** showed mitral valve prolapse. **B** showed thickened, roughened mitral valve leaflets. **C** showed severe mitral regurgitation

The patient's clinical examination revealed an audible blowing murmur in the precordial area, normal limb muscle strength (5/5) and bilaterally diminished acupuncture sensation below the T12 dermatome. Proprioception was intact. Meningeal irritation was negative. cranial nerves were not involved. Laboratory test results showed: complete blood count (WBC 11.35*10^9^/L, NEUT 8.28*10^9^/L), ESR 20.00 mm/h, cerebrospinal fluid leukocyte 101*10^6^/L, LDH 45.35U /L, protein levels did not rise and glucose levels did not fall. Intracranial pressure was 210 mmH2O. MRI showed patchy long T1 and long T2 signals in the right parietal lobe, left frontal lobe and bilateral occipital lobes (Fig. 1). MRI showed diffuse edema of spinal cord C1-T1 with an enhanced signal on T2WI (Fig. 2). The patient was initially considered to have a central nervous system infection. It was necessary to differentiate between a nervous system tumor and a nervous system demyelinating disease. Contrast-enhanced MRI showed multiple ring-enhancing lesions in the brain and ring-enhancing images of C4-C5 in the spinal cord (Fig. 3). Magnetic resonance spectroscopy indicated nerve cell destruction, hypoxia and necrosis. And tumor was excluded (Fig. 4). Echocardiography reported thickened, roughened mitral valve leaflets, possible vegetations, mitral valve prolapse, and severe mitral regurgitation (Fig. 5). Test results for other infections were negative. *Streptococcus anginosus* was obtained from blood culture on the 8th day of admission. The patient was diagnosed with a central system infection caused by subacute infective endocarditis. According to the drug susceptibility results, intravenous ceftriaxone combined with linezolid anti-infective treatment continued for 24 days until the patient was discharged from the hospital. After discharge, the patient was adjusted to oral linezolid and cefixime anti-infective treatment.

**Fig. 6:**
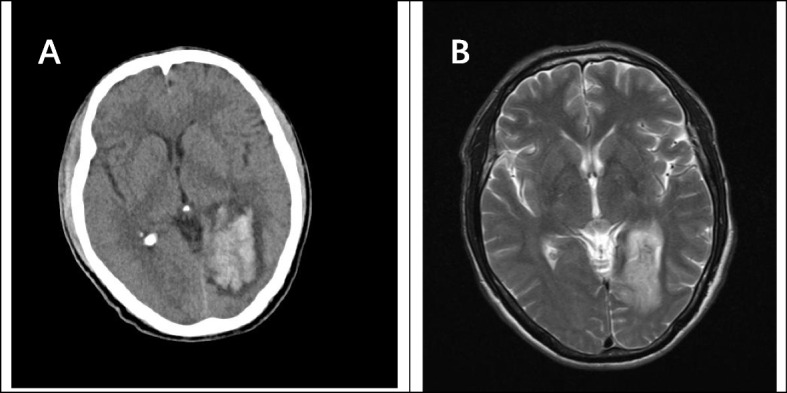
Imaging of hemorrhage in brain abscess. **A** Left occipital lobe cerebral hemorrhage focused on CT. **B** Corresponding brain abscess site before upper cerebral hemorrhage on MRI

During hospitalization, the patient developed nausea and vomiting. The vomit was stomach contents, not projectile vomiting. Emergency head computer tomography showed bleeding in the left occipital lobe (Fig. 6). This was considered secondary intracerebral hemorrhage due to bacterial invasion of blood vessels. After monitoring blood pressure and micro pumping nimodipine to relieve vasospasm, the patient did not experience similar symptoms.

One month after discharge from the hospital, the re-examination of the MRI of the brain and spinal cord showed that the range of lesions was smaller than before (Figs. 1, 2 and 3). The contrast-enhanced MRI improved. The patient's blurred vision and head and neck pain improved compared with before, but left lower limb numbness remained.

## Discussion

ISCA is a rare entity, even rarer when it is associated with a brain abscess. Our review of the literature suggests that ISCA mainly involves the cervical cord [1–3, 5–8]. An ISCA typically presents with progressive back pain with fever followed by neurological deficits, and it can also present with acute neurological deficits similar to episodes of transverse myelitis [3, 6]. The specific symptoms are location-dependent and rapidly progressive [8]. But sometimes the clinical manifestations may progress insidiously [9].The mechanisms of infection include hematogenous dissemination, contiguous spread from adjacent infection or infected dermal sinus, direct penetrating trauma, or septic emboli [10]. Many organisms can cause intramedullary abscesses, including *Mycobacterium tuberculosis* in the developing world and gram-positive cocci in the developed world [3]. In terms of clinical manifestations, the patient presented to our hospital complaining of head and neck pain and numbness in both lower extremities for 7 days. There was no fever at the time of admission, and a fever ≥ 38.0℃ occurred after admission. The blood culture was *streptococcus anginosus*. *Streptococcus anginosus* group bacteria are gram-positive organisms that are part of the oral and gastrointestinal microbiome [11]. They are common pathogens in ISCA. *Str. viridans* was seen in 25% of all streptococcal ISCA and is a common pathogen in subacute bacterial endocarditis [2]. Subacute infective endocarditis progresses insidiously, with no fever in the early stage. Therefore, patients with no fever in the early stage should also be alert to infective endocarditis.

Contrast-enhanced MRI is the method of choice when intramedullary or brain abscesses are suspected [3, 12]. Typical MRI features of intramedullary and brain abscesses are hypointensity on T1WI, the increased signal on T2WI and peripheral contrast enhancement with gadolinium [6, 13]. The hyperintensity on T2-weighted images gradually subsided as the infection resolved after treatment [14]. The images of our patients were similar to typical images. After treatment, the hyperintensity range on T2WI was reduced. It is worth mentioning that our case suffered from nausea and vomiting during hospitalization. Computer tomography showed an intracerebral hemorrhage at the location of the left occipital brain abscess. At present, cerebral hemorrhage caused by brain abscess is related to many influencing factors, but the pathogenesis is not very clear. The authors support the idea that a strong inflammatory response destroys fragile new blood vessels, leading to vascular rupture and secondary cerebral hemorrhage [15].

For treatment and prognosis, a reasonable empiric regimen in an immunocompetent patient without recent instrumentation would be vancomycin, ceftriaxone, and metronidazole. This regimen can cover gram-positive bacteria (including methicillin-resistant Staphylococcus aureus), gram-negative bacteria and anaerobes [16]. Our patient was given ceftriaxone combined with linezolid based on blood culture susceptibility results. Both of them not only have excellent anti-streptococcal activity, but also have the characteristics of strong tissue penetration and the ability to cross the blood–brain barrier, which can achieve higher cerebrospinal fluid concentration [3, 7]. The optimal duration of treatment has not been established. Patients should be followed closely with serial neurological examinations and MRIs. Mortality has recently declined due to imaging techniques and antibiotic use but remains at 4%. Neurological sequelae occur in 60% of surviving patients [4]. Patients with persistent bacteremia and a combined brain and spinal cord abscess should be treated with surgery, but in patients with hemorrhagic stroke, surgery should be delayed for at least 4 weeks and reimaging should be performed before surgery [17, 18]. At the follow-up 1 month later, our patient's symptoms and imaging findings were improved but left lower extremity numbness remained. The patient is advised to see a cardiovascular surgeon for surgical evaluation.

In conclusion, although ISCA is a rare entity, we need to increase awareness and vigilance of ISCA. Early diagnosis and rapid use of broad-spectrum antibiotics are the keys to halting disease progression and reducing mortality. When the patient's symptoms, signs or imaging suggest an ISCA, regardless of whether there are symptoms such as fever and headache, blood culture, spinal cord, head MRI and echocardiography should be completed early to identify whether the patient is complicated with bacteremia and brain abscess, and then identify whether the cause is infective endocarditis.

## Data Availability

The datasets used and analyzed during the current study are available from the corresponding author on reasonable request.

## Abbreviations

ISCA: Intramedullary spinal cord abscess
MRI: Magnetic resonance imaging

## References

[1] Vo DT, Cravens GF, Germann RE (2016). Streptococcus pneumoniae meningitis complicated by an intramedullary abscess: a case report and review of the literature. J Med Case Rep.

[2] Terterov S, Taghva A, Khalessi AA (2011). Intramedullary abscess of the spinal cord in the setting of patent foramen ovale. World Neurosurg.

[3] Cerecedo-Lopez CD, Bernstock JD, Dmytriw AA (2022). Spontaneous intramedullary abscesses caused by streptococcus anginosus: two case reports and review of the literature. Bmc Infect Dis.

[4] Kurita N, Sakurai Y, Taniguchi M (2009). Intramedullary spinal cord abscess treated with antibiotic therapy–case report and review. Neurol Med-Chir.

[5] Bakhsheshian J, Kim PE, Attenello FJ (2017). Intramedullary cervical spinal cord abscess. World Neurosurg.

[6] Hood B, Wolfe SQ, Trivedi RA (2011). Intramedullary abscess of the cervical spinal cord in an otherwise healthy man. World Neurosurg.

[7] Arnaiz-Garcia ME, Gonzalez-Santos JM, Lopez-Rodriguez J (2015). Intramedullary cervical abscess in the setting of aortic valve endocarditis. Asian Card Thorac an.

[8] Erlich JH, Rosenfeld JV, Fuller A (1992). Acute intramedullary spinal cord abscess: case report. Surg Neurol.

[9] Akhaddar A, Boulahroud O, Boucetta M (2011). Chronic spinal cord abscess in an elderly patient. Surg Infect.

[10] Desai KI, Muzumdar DP, Goel A (1999). Holocord intramedullary abscess: an unusual case with review of literature. Spinal Cord.

[11] Sitkiewicz I (2018). How to become a killer, or is it all accidental? Virulence strategies in oral streptococci. Mol Oral Microbiol.

[12] Sonneville R, Ruimy R, Benzonana N (2017). An update on bacterial brain abscess in immunocompetent patients. Clin Microbiol Infec.

[13] Cantiera M, Tattevin P, Sonneville R (2019). Brain abscess in immunocompetent adult patients. Rev Neurol-France.

[14] Murphy KJ, Brunberg JA, Quint DJ (1998). Spinal cord infection: myelitis and abscess formation. Am J Neuroradiol.

[15] Kaplan M, Topsakal C, Cihangiroglu M (2006). Hemorrhage into the brain abscess cavity with fallot's tetralogy. Pediatr Neurosurg.

[16] Nau R, Sorgel F, Eiffert H (2010). Penetration of drugs through the blood-cerebrospinal fluid/blood-brain barrier for treatment of central nervous system infections. Clin Microbiol Rev.

[17] Hubers SA, DeSimone DC, Gersh BJ (2020). Infective endocarditis: a contemporary review. Mayo Clin Proc.

[18] Pettersson GB, Coselli JS, Pettersson GB (2017). 2016 the american association for thoracic surgery (aats) consensus guidelines: surgical treatment of infective endocarditis: executive summary. J Thorac Cardiov Sur.

